# Next-Generation Sequencing Reveals Increased Anti-oxidant Response and Ecdysone Signaling in STAT Supercompetitors in *Drosophila*

**DOI:** 10.1534/g3.119.400345

**Published:** 2019-06-21

**Authors:** Poojitha Sitaram, Sean Lu, Sneh Harsh, Salvador C. Herrera, Erika A. Bach

**Affiliations:** Department of Biochemistry and Molecular Pharmacology, New York University School of Medicine, NY

**Keywords:** JAK/STAT, cell competition, wing imaginal disc, RNA-seq, Myc

## Abstract

Cell competition is the elimination of one viable population of cells (the losers) by a neighboring fitter population (the winners) and was discovered by studies in the *Drosophila melanogaster* wing imaginal disc. Supercompetition is a process in which cells with elevated JAK/STAT signaling or increased Myc become winners and outcompete wild-type neighbors. To identify the genes that are differentially regulated in STAT supercompetitors, we purified these cells from *Drosophila* wing imaginal discs and performed next-generation sequencing. Their transcriptome was compared to those of control wing disc cells and Myc supercompetitors. Bioinformatics revealed that STAT and Myc supercompetitors have distinct transcriptomes with only 41 common differentially regulated genes. Furthermore, STAT supercompetitors have elevated reactive oxygen species, an anti-oxidant response and increased ecdysone signaling. Using a combination of methods, we validated 13 differentially expressed genes. These data sets will be useful resources to the community.

Competitive interactions between cells are ubiquitous, and the resolution of such interactions regulates a broad range of biological processes (Amoyel and Bach 2014; Johnston 2014; Clavería and Torres 2016; Baker 2017; Nagata and Igaki 2018). Cell competition was discovered by studies in developing epithelia of *Drosophila* (Morata and Ripoll 1975; Simpson 1979; Simpson and Morata 1981). Animals harboring mutations in ribosomal genes were viable in a homotypic environment but were eliminated when grown in a heterotypic environment with more robust wild-type cells. The eliminated cells were referred to as ‘losers’, and the cells that outcompete them were termed ‘winners’. This context-dependent elimination of a viable cell population was termed ‘cell competition’. Since these pioneering studies, additional types of competitive interactions have been reported. These include the context-dependent elimination of viable cells with decreased metabolism or signal transduction, or with aberrant polarity (Moreno *et al.* 2002; Brumby and Richardson 2003; Pagliarini and Xu 2003; De La Cova *et al.* 2004; Moreno and Basler 2004; Igaki *et al.* 2006; Tyler *et al.* 2007; Neto-Silva *et al.* 2010; Tamori *et al.* 2010; Ziosi *et al.* 2010; Ohsawa *et al.* 2011; Vincent *et al.* 2011; Rodrigues *et al.* 2012; Schroeder *et al.* 2013). Importantly, cell competition is conserved in mammals and is triggered by differences in common factors like Myc. In mammals, competitive interactions between cells occur during embryogenesis and in adulthood and are important in both regenerative and homeostatic processes (Oliver *et al.* 2004; Clavería *et al.* 2013; Sancho *et al.* 2013; Martins *et al.* 2014; Villa Del Campo *et al.* 2014; Villa Del Campo *et al.* 2016; Díaz-Díaz *et al.* 2017; Liu *et al.* 2019).

Wild-type cells can become losers and be eliminated when confronted by cells with elevated activity or levels of certain proto-oncogenic pathways, including JAK/STAT, Myc, Wingless (Wg)/Wnt or Yorkie (Yki)/YAP (De La Cova *et al.* 2004; Moreno and Basler 2004; Neto-Silva *et al.* 2010; Ziosi *et al.* 2010; Vincent *et al.* 2011; Rodrigues *et al.* 2012). The elimination of wild-type cells by cells with higher levels of proto-oncogenic factors has been termed “supercompetition”. Winners eliminate less fit cells through direct contact and by the production of short-range soluble factors that kill losers at a distance (De La Cova *et al.* 2004; Li and Baker 2007; Martin *et al.* 2009; Ohsawa *et al.* 2011; Rodrigues *et al.* 2012; Ballesteros-Arias *et al.* 2014; Meyer *et al.* 2014; Kucinski *et al.* 2017; Yamamoto *et al.* 2017; Alpar *et al.* 2018). While the identities of these latter soluble factors are largely unknown, recent work from the Johnston lab has shown that Myc supercompetitors secrete serine proteases to create a local burst of active Spätzle (Spz), triggering Toll signaling and consequently apoptosis in less fit neighbors (Alpar *et al.* 2018). However, it is not clear whether other kinds of supercompetitors eliminate wild-type cells through a similar mechanism.

We previously reported that clones with higher levels of JAK/STAT signaling (termed STAT supercompetitors) eliminated neighboring wild-type cells by non-autonomously inducing *hid*-dependent apoptosis (Rodrigues *et al.* 2012). To gain insights into how STAT supercompetitors acquire their competitive advantages, we performed next generation sequencing on FACS-purified STAT supercompetitors and compared their transcriptome to that of FACS-purified Myc supercompetitors and FACS-purified control cells from wing imaginal discs. Analysis of these data sets reveal 1004 genes (*P* < 0.05) that were differentially regulated in STAT supercompetitors, including known JAK/STAT target genes *Socs36E*, *chinmo* and *domeless* (*dome*) (Flaherty *et al.* 2009; Flaherty *et al.* 2010; Herrera and Bach 2019). Additionally, 328 genes (*P* < 0.05) were differentially regulated in Myc supercompetitors, including known Myc targets *Nop60B*, *nop5* and *Tif-1A* (Grewal *et al.* 2005). There was limited overlap between these data sets with only 41 genes differentially regulated in both STAT and Myc supercompetitors, 24 upregulated in both conditions and 17 downregulated in both conditions. Of the differentially regulated genes in STAT supercompetitors, 210 had STAT binding sites in regulatory regions, suggesting that they could be directly regulated by JAK/STAT signaling. These include known JAK/STAT target genes, *Socs36E*, *chinmo* and *dome*, as well as several in the ecdysone signaling pathway, including the *Ecdysone receptor* (*EcR*) and its targets *Ecdysone-induced protein 75B (Eip75B)*, *ftz-f1*, and *Ecdysone-inducible gene E1 (ImpE1)*. We validated 13 upregulated genes, 10 of which were increased only in STAT supercompetitors and 3 of which were upregulated in both STAT and Myc supercompetitors. Finally, we established a quantitative assay for supercompetition that can be used in future studies to test the functional significance of differentially regulated genes.

## Materials And Methods

### Fly Stocks

We used *dpp-gal4*, *UAS-gfp/TM6B*, *Tb* (a gift of Laura Johnston, Columbia University Medical Center, NY, USA), *UAS-hop* and *UAS-Myc* for FACS. We crossed *y*, *w*; *act > y+>gal4*, *UAS-gfp* to *y*, *w*, *hs-flp^122^*; *UAS-Dcr-2*; *+/+* to make GFP flip-out (GFP FO) clones or to *y*, *w*, *hs-flp^122^*; *UAS-Dcr-2*; *UAS-hop/TM6B* to make Hop flip-out (Hop FO) clones. We used *PBac[cnc-EGFP.S]VK00037* (Bloomington *Drosophila* Stock Center (BDSC), BL-38631) to monitor endogenous expression of Nrf2 (*Drosophila* Cap-n-collar (Cnc)). We used *UAS-Stat92E^HMS00035^* RNAi (BDSC, BL-33637) (termed *STAT-i*) to deplete *Stat92E* from GFP FO or Hop FO clones in the cell competition assay (see below). We maintained crosses at 25° on standard food and in a 12-hour light/dark incubator.

### Time to pupariation

To determine the time to pupariation, we used a protocol published by the Léopold lab (Colombani *et al.* 2015). We made 4-hour embryo collections from the cross *dpp-gal4*, *UAS-gfp/TM6B*, *Tb* x *Ore^R^* and the cross *dpp-gal4*, *UAS-gfp/TM6B*, *Tb* x *UAS-hop*/*TM6B*, *Tb*. We collected first instar larvae at 24 hr after egg deposition (AED) and reared 30 larvae per vial on standard food at 25°. At 90 hr AED, we monitored the time to pupariation every 6 hr. We calculated the average time to pupariation and the standard error of the mean for 30 *dpp-gal4*, *UAS-gfp/+* and 30 *dpp-gal4*, *UAS-gfp/UAS-hop* larvae using Excel.

### Flow cytometry and RNA isolation

We crossed *dpp-gal4*, *UAS-gfp/TM6B*, *Tb* to *UAS-hop* or to *UAS-Myc*. From non-*Tb* larvae, we dissect at least 60 third instar wing discs per genotype in triplicate at approximately 110-115 hr AED. The cells were dissociated and the GFP-positive cells were sorted by the Cytometry and Cell Sorting Core at NYU Langone Medical Center using a Sony SY3200 cell sorter per the protocol described in (De La Cruz and Edgar 2008). The sorted cells represent GFP-positive control cells from the *dpp* domain (referred to as “GFP” samples), GFP-positive cells from the *dpp* domain that had ectopic JAK/STAT signaling as a result of mis-expressing Hop (referred to as “Hop” samples) or GFP-positive cells from the *dpp* domain that had elevated Myc levels as a result of mis-expressing Myc (referred to as “Myc” samples). We isolated RNA from the sorted cells using TRIzol reagent (Ambion) and then purified the RNA using RNeasy Mini Kit (Qiagen) per the manufacturer’s instructions.

### Quantitative PCR (qPCR)

We performed qPCR using the SYBR Green PCR Mix (Applied Biosystems) protocol and a real-time PCR machine (ABI 7900HT) from Applied Biosystems. We isolated RNA as described above and synthesized cDNA using the SuperScript Reverse Transcriptase II kit (Invitrogen) per the manufacturer’s instructions. We measured the cDNA concentration using a Nanodrop ND-1000. We used 3 ng of cDNA per sample per reaction, 5 µM of each primer, and 1x SYBR. We performed the qPCR in triplicates per primer per sample. We normalized to *tubulin* (*β-tub56d*). The data were graphed using Excel, and statistical significance was determined using Student’s *t*-test in Excel. We used the following primers:

*Socs36E* F: GCTGCCAGTCAGCAATATGT and R: GACTGCGG-CAGCAACTGT

*dome* F: CGGACTTTCGGTACTCCATC and R: GATCGATCAT-CGCCGAGTT

*Tif-1A* F: GTAGCGAAGAACAGCGAAGG and R: AATTGCAC-ATGATGCGTGTT

*β-tub56d*: F: CTCAGTGCTCGATGTTGTCC and R: GCCAAGG-GAGTGTGTGAGTT

### RNA-seq

The RNA sequencing was performed by the Genome Technology Center at the NYU Langone Medical Center. 10 ng of total RNA was used for library prep, and cDNA was amplified by using Nugen Ovation RNA-Seq System V2 kit (Part No. 7102-32), 100 ng of Covaris-fragmented cDNA were used as input to prepare the libraries, using the Ovation Ultralow Library system (Nugen, Part 0330-32), and amplified by 10 cycles of PCR. The samples were mixed into two pools and run in two 50-nucleotide paired-end read rapid run flow cell lanes on the Illumina HiSeq 2500 sequencer.

### Bioinformatics

Bioinformatic analysis was performed by the Applied Bioinformatics Laboratories at NYU Langone Medical Center. Sequencing results were demultiplexed and converted to FASTQ format using Illumina Bcl2FastQ software. Reads were aligned to the dm6 release of the *Drosophila melanogaster* genome using the splice-aware STAR aligner. PCR duplicates were removed using the Picard toolkit (http://broadinstitute.github.io/picard). The HTSeq package (Love *et al.* 2014) was utilized to generate counts for each gene based on how many aligned reads overlap its exons. These counts were then used to test for differential expression using negative binomial generalized linear models implemented by the DESeq2 R package (Anders *et al.* 2015). The adjusted p-value (p^adj^) was generated by using the False Discovery Rate with the Benjamini-Hochberg method. Scatter (Volcano) plots were generated using Stata 15.1 (StataCorp LLC, College Station TX). For Table S1, we chose a cut-off at fold change ≥ 1.5 (corresponding to log_2_(fold change) 0.58) and an adjusted p-value < 0.05 because this data set would include known JAK/STAT target genes *dome*, *Socs36E* and *zfh2* (Flaherty *et al.* 2009; Ayala-Camargo *et al.* 2013). We then decided to reciprocally apply this cut-off (fold change ≤0.667 corresponding to log_2_(fold change) -0.58) and an adjusted p-value < 0.05 to the downregulated genes. We then applied these cut-offs for differentially-regulated genes in the Myc supercompetitor RNA-seq in Table S2.

### Genome-wide analysis of Stat92E binding sites

We obtained a positional weight matrix (PWM) for Stat92E from Jaspar (http://jaspar.genereg.net/matrix/MA0532.1/) (Khan *et al.* 2018). We used a web-based program PWMScan (https://ccg.epfl.ch//pwmtools/pwmscan.php (Ambrosini *et al.* 2018)) to search the *Drosophila* genome for Stat92E binding sites that matched that PWM with a stringent p value less than 1x10^−5^ (recommended by developers of the PWMScan website (Ambrosini *et al.* 2018)). We then compared the list of locations of Stat92E binding sites with the list of genes and their locations (https://genome.ucsc.edu/cgi-bin/hgTables?command=start). We report in Tables S5 and S6 differentially regulated genes in STAT supercompetitors with at least one Stat92E binding site in non-coding regions, defined as 1,500 bps upstream of the transcription start site, introns, and 1,500 bps downstream of the termination sequence.

### Riboprobe synthesis

We used these EST clones from the *Drosophila* Genomics Resource Center (DGRC) for riboprobe synthesis: *hop* (*RH47993*); *Socs36E* (*SD04308*); *Ama* (*LD39923*); *dilp8* (*IP06570*); *Mmp1* (*RE62222*); *sas* (*LD44801*); *edl* (*LD15796*); *ftz-f1* (*LD15303*); *ImpE1* (*IP15635*); *mld* (*SD03914*); *Mpcp2* (*RE67391*); *mnd* (*LD25378*). RNA probes were designed against the contiguous cDNA sequence of differentially expressed genes. The DGRC probes were synthesized using 1-5 µg of linearized plasmid in a 20 µl transcription reaction mix. We used a digoxigenin (DIG)-labeling kit (Roche) per the manufacturer’s instructions. The resulting labeled riboprobes were ethanol precipitated and re-suspended in 100 µl of hybridization buffer (HB4) containing 50% formamide, 5x saline sodium citrate (SSC), 50 µg/ml heparin, 0.1% Tween-20 and 5 mg/ml of Tortula Yeast RNA extract.

### in situ hybridization

Wandering, mid-third instar wing discs were dissected in cold 1x phosphate buffered saline (PBS) and fixed in 4% paraformaldehyde for 20 min. They were subsequently washed three times in 1x PBS 0.1% Tween 20 (1xPBS-T) for 10 min, rehydrated in decreasing concentrations of methanol and treated with 10 µg/ml proteinase K for 5 min. They were then fixed in 4% paraformaldehyde for 20 min, washed in 1xPBS-T, treated with acetylation solution (9.25 g triethanolamine HCL +1.12 ml 10N NaOH in 500 ml H_2_O +12.5 µl acetic anhydride) for 10 min and prehybridized for 1 hr at 65° in HB4. The discs were hybridized overnight in 100 µl of HB4 and 1 µl of the riboprobe that had already been denatured at 80° for 10 min in HB4 and then put on ice. After hybridization, the discs were washed two times for 25 min in a buffer containing 50% formamide, 50% 2x SSC with 0.1% Tween-20. They were rinsed in 1x PBS-T at room temperature three times for 10 min. Subsequently, they were incubated for 2 hr with anti-DIG (Roche; diluted 1:2000) and then washed three times for 10 min in 1x PBS-T. After this, they were rinsed once and washed for 5 min in alkaline phosphate buffer pH 9.5 containing 0.1 M NaCl, 0.05 M MgCl_2_, 0.1 M Tris (pH 9.5) and 0.1% Tween-20. The reaction was developed by adding 40 µl of NBT/BCIP stock solution to 2 ml of 1x PBS.

### Antibody staining, ROS detection and TUNEL

Immunofluorescence was performed as described in (Ekas *et al.* 2006). We used rabbit anti-Stat92E (1:500, (Flaherty *et al.* 2010)), rabbit anti-Dcp-1 (1:100) (Cell Signaling), rabbit anti-GFP (1:500) (Invitrogen), mouse anti-Patched Apa1 (1:10) (Development Studies Hybridoma Bank (DSHB)), mouse anti-Ptp10D 8B22F5 (1:5) (DSHB), Alexa647 Phalloidin (Invitrogen), fluorescent secondary antibodies at 1:250 (Jackson Laboratories), and Vectashield (Vector labs). We monitored ROS using CellROX Deep Red Reagent (Invitrogen) and followed the protocol in (Santabárbara-Ruiz *et al.* 2015). Briefly, we dissected third instar wing discs in Schneider’s medium containing 5 μM CellROX Deep Red Reagent and incubated the discs for 15 min, followed by three washes in Schneider's medium. We then immediately analyzed the samples on a Zeiss LSM 510 confocal microscope at 25x. The samples were protected from light throughout the experiment. For terminal deoxynucleotidyl transferase dUTP nick end labeling (TUNEL), we first stained with the pSTAT primary antibody and then with Cy5 Donkey anti-Rabbit secondary (1:250, Jackson Immunochemicals). After washing the fluorescent secondary antibody in 1x PBS, we prepared the TUNEL reaction (Roche # 12156792910) by adding the enzyme solution to label solution in a 1:10 dilution, with enough volume prepared to load 50 µl per sample. The solution was mixed well and kept on ice. We added 50 μL of label solution alone to the negative control. We added 50 μL of TUNEL reaction mixture to each sample tube. We incubated the negative control and the experimental samples at 37° for 1 hr. We then rinsed all samples twice with 1x PBS for 1 min. The samples were then mounted in Vectashield. We collected fluorescent images at 25x magnification using a Zeiss LSM 510 confocal microscope.

### Quantitative assay for supercompetition

We made 4-hour timed embryo collections. Clones expressing GFP alone (labeled GFP flip-out (FO)) or GFP and Hop (labeled Hop FO) were randomly induced by *hs-flp* for 10 min at 48 hr AED. Wing imaginal discs were dissected at 72 hr after clone induction, and they were fixed, stained and imaged as described above. We used Image J to outline the flip-out clones and then to draw a second line at a distance of 10-cell diameters from the clone boundary using these values: 512 pixels = 509.12µm; 1 cell = 5µm; 1cell = 5pixels. We then counted the number of apoptotic (Dcp-1-positive) cells within the area delimited by the two lines. At least 15 clones per genotype were analyzed. The data were graphed using Excel, and statistical significance was determined using Student’s *t*-test in Excel.

### Data availability statement

Strains and plasmids are available upon request. We obtained a PWM for Stat92E from Jaspar (http://jaspar.genereg.net/matrix/MA0532.1/) (Khan *et al.* 2018). We used a web-based program PWMScan (https://ccg.epfl.ch//pwmtools/pwmscan.php (Ambrosini *et al.* 2018)) to search the *Drosophila* genome for Stat92E binding sites. Table S1 contains the list of genes that are differentially up- or downregulated (fold change 1.5 for upregulated genes, p-value < 0.05 and 0.667 for downregulated genes, p-value < 0.05) in STAT supercompetitors. Table S2 contains the list of genes that are differentially up- or downregulated (fold change 1.5, p-value < 0.05 for upregulated genes and 0.667 for downregulated genes, p-value < 0.05) in Myc supercompetitors. Table S3 contains the list of genes that are differentially upregulated (fold change 1.5, p-value < 0.05) in both STAT and Myc supercompetitors. Table S4 contains the list of genes that are differentially-downregulated (fold change 0.667, p-value < 0.05) in both STAT and Myc supercompetitors. Table S5 contains the list of genes that are differentially-upregulated in STAT supercompetitors that contain at least one STAT binding site. Table S6 contains the list of genes that are differentially-downregulated in STAT supercompetitors that contain at least one STAT binding site. The RNA-seq data in this study have been deposited at NCBI’s Gene Expression Omnibus (Edgar *et al.* 2002) and are accessible through GEO Series accession number (GSE130993) (https://www.ncbi.nlm.nih.gov/geo/query/acc.cgi?acc=GSE130993). Supplemental material available at FigShare: https://doi.org/10.25387/g3.8242655.

## Results

### Ectopic activation of the JAK/STAT pathway induces cell competition

**Figure 1:**
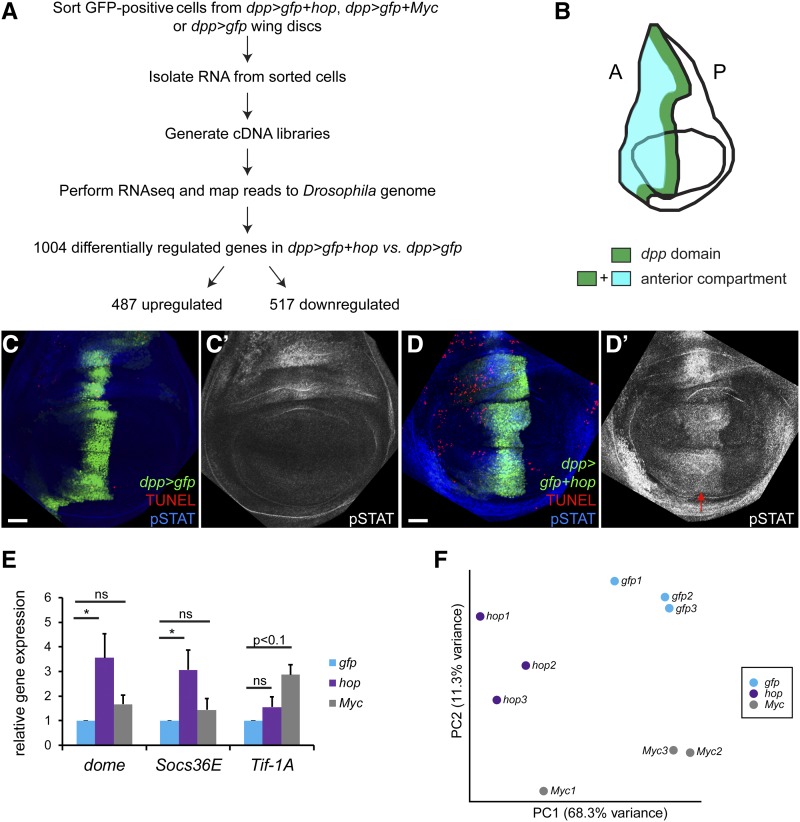
STAT supercompetitors outcompete wild-type neighbors. (A) Work-flow of the RNA-seq. Briefly, we purified GFP-positive cells from *dpp > gfp* (control), *dpp > gfp+hop* (STAT supercompetitiors) or *dpp > gfp+Myc* (Myc supercompetitors) wing discs. We isolated total RNA from these cells and generated cDNA libraries, which were used for the RNA-seq. The reads were mapped to the *Drosophila* genome (dm6). 1004 genes were differentially expressed in *dpp > gfp+hop* cells compared to *dpp > gfp* cells, with 487 upregulated and 517 downregulated. (B) Cartoon of a third instar wing imaginal disc. The *dpp* expression domain (green stripe) resides within the anterior compartment (blue area). (C-D) In control *dpp > gfp* discs, few cells were undergoing programmed cell death (C, red cells) in either the anterior or posterior compartment, and Stat92E is not ectopically upregulated in the *dpp* domain (C’, white). By contrast, in *dpp > gfp+hop* discs, there were substantially more apoptotic cells in the anterior compartment (D, red cells), due to the competitive stress inflicted by STAT winners residing in the *dpp* stripe. The ectopic expression of Hop in the *dpp* domain ectopically activates Stat92E (D’, white). GFP is in green, TUNEL marking apoptotic cells is in red; activated Stat92E (labeled “pSTAT”) is in blue. Scale bar indicates 50 µM. (E) Quantitative PCR analysis of RNA isolated from FACS-purified, *dpp*-domain wing cells reveals that JAK/STAT targets *dome* (*P* < 0.05) and *Socs36E* (*P* < 0.05) are significantly increased in *dpp > hop* samples (purple) but not *dpp > Myc* samples (gray) compared to control *dpp > gfp* (blue) and that the Myc target *Tif-1A* (*P* < 0.1) is significantly increased in *dpp > Myc* samples (gray) but not in *dpp > hop* samples (purple) compared to controls (blue). The results were averages of 4 independent biological replicates. * *P* < 0.05; “ns” means not significant. (F) Principal component analysis for *gfp* (blue), *hop* (purple) and *Myc* (gray) triplicate samples. Genotypes (C) *w/w*; *+/+*; *dpp-gal4*, *UAS-gfp/+* (D) *w/w*; *+/+*; *dpp-gal4*, *UAS-gfp/UAS-hop* (E,F) *w/w*; *+/+*; *dpp-gal4*, *UAS-gfp/+* (gfp), *w/w*; *+/+*; *dpp-gal4*, *UAS-gfp/UAS-hop *(hop), *w/w*; *+/+*; *dpp-gal4*, *UAS-gfp/UAS-Myc* (Myc).

Cell competition is induced in the *Drosophila* wing disc when neighboring populations differ in levels of JAK/STAT activity (Rodrigues *et al.* 2012). To identify JAK/STAT pathway targets that may regulate cell competition, we carried out RNA-seq analysis of winners with elevated Stat92E activity, with elevated Myc, or GFP control cells (Figure 1A). We induced cell competition in the anterior compartment of the wing disc by mis-expressing the *Drosophila* Janus Kinase, Hopscotch (Hop), using *UAS-hop* and *dpp-gal4*, *UAS-gfp* transgenes in the anterior midline of the disc (Figure 1B,D). Ectopic mis-expression of Hop autonomously activates STAT, as evidenced by stabilized Stat92E protein within cells in the *dpp* domain in *dpp > gfp+hop* discs (hereafter referred to as *dpp > hop*). These cells, termed STAT supercompetitors or STAT winners, induce the apoptotic death of wild-type neighboring cells as evidenced by increased TUNEL staining in anterior cells outside of the *dpp* domain in *dpp > hop* discs (Figure 1D), consistent with our prior work (Rodrigues *et al.* 2012). As cell competition does not cross compartment boundaries (Morata and Ripoll 1975; De La Cova *et al.* 2004), ectopic activation of JAK/STAT signaling in the anterior midline does not induce cell death of wild-type cells located in the posterior compartment (Figure 1D). GFP-positive cells from *dpp > gfp* wing discs served as the control. Mis-expression of *gfp* in the *dpp* domain does not activate STAT (Figure 1C), nor does it induce competitive death of wild-type neighbors in the anterior domain (Figure 1C). Mild ectopic activation of the JAK/STAT pathway in the *dpp* domain does not perturb developmental timing. The time of pupariation of *dpp*>*hop* larvae is 120 ± 0.7 hr (n = 30) at 25° compared to 124 ± 0.5 hr for *dpp*>*gfp* controls (n = 30) under the same conditions.

The Johnston lab has previously reported that expression of Myc in the *dpp* domain induces competitive interactions between the cells with elevated Myc, termed Myc supercompetitors, and neighboring wild-type cells in the anterior compartment. These interactions result in *hid*-dependent death of the wild-type neighbors located within an ∼8 cell diameter distance of the Myc supercompetitors (De La Cova *et al.* 2004).

### Next-generation sequencing of FACS-purified STAT or Myc supercompetitors

We isolated viable STAT supercompetitors (*dpp > hop*), Myc supercompetitors (*dpp > gfp+Myc*, referred *to as dpp > Myc*) and control *dpp > gfp* cells by flow cytometry based on their lack of propidium iodide uptake and their expression of GFP in the *dpp* domain. To confirm that the isolated cells had the correct genotype, we performed quantitative PCR analysis of RNA extracted from sorted cells. This revealed significantly increased expression of known JAK/STAT targets *dome* and *Socs36E* in STAT supercompetitors (*P* < 0.05) over control cells and significantly increased expression of the Myc target *Tif-1A* in Myc supercompetitors (*P* < 0.1) over control cells (Figure 1E and (Grewal *et al.* 2005; Flaherty *et al.* 2009). The isolated RNA was processed for expression profiling, and the sequencing was performed using Illumina HiSeq2500 Paired-End 50 Cycle Flow Cell. Principal Component Analysis revealed that control (labeled *gfp*), *hop* and *Myc* samples were distinct clusters (Figure 1F).

**Figure 2:**
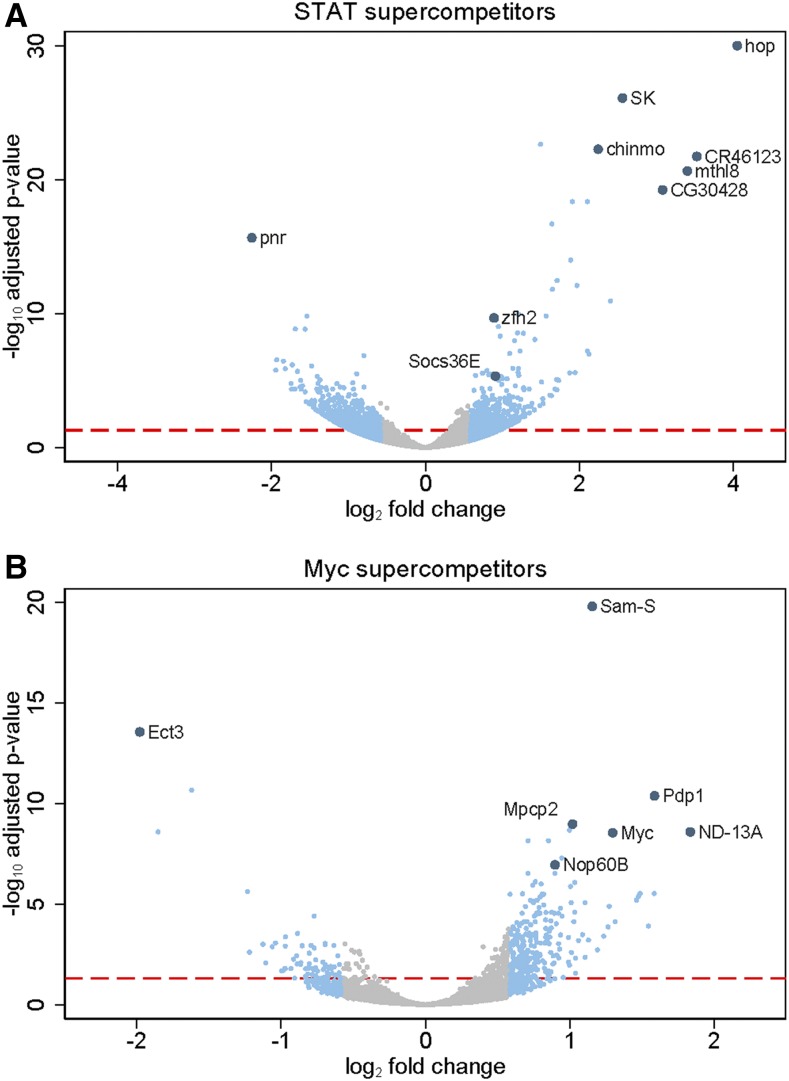
Volcano plots of gene expression in STAT and Myc supercompetitors. (A,B) Scatter (Volcano) plot for genes in STAT supercompetitors (*dpp > hop*) compared to controls (*dpp > gfp*) in A and for genes in Myc supercompetitors (*dpp > Myc*) compared to controls (*dpp > gfp*) in B. The x-axis is the log_2_ of the fold change and the y-axis is the negative log_10_ of the adjusted p-value. Gray circles indicate genes with log_2_(fold change) between -0.58 and 0.58 (corresponding to fold change between 0.667 and 1.5). Blue circles indicate genes with log_2_(fold change) ≤-0.58 and ≥0.58 (corresponding to fold change ≤0.667 and ≥1.5). The larger blue circles indicate the mis-expressed genes (*hop* in A and *Myc* in B), known target genes (*chinmo*, *zfh2*, *Socs36E*, *pnr* in A and *Nop60B* in B) or highly differentially-regulated genes in the data sets (*SK*, *CR46123*, *mthl8*, and *CG30428* for A and *Pdp1*, *ND-13A*, *Mpcp2* and *Ect3* in B). Genotypes (A) *w/w*; *+/+*; *dpp-gal4*, *UAS-gfp/UAS-hop* (B) *w/w*; *+/+*; *dpp-gal4*, *UAS-gfp/UAS-Myc*.

For the bioinformatic analyses, we chose an arbitrary cut-off of fold change ≥ 1.5 (corresponding to log_2_(fold change) 0.58) and an adjusted p-value < 0.05 for upregulated genes because this set of 487 genes includes known JAK/STAT target genes (see below and Materials and Methods). As expected, *hop* was the transcript with the highest fold change (16.6 fold, *P* < 4.73 × 10^−180^) in the STAT supercompetitors (Table 1, Figure 2A) and served as the internal control for this study. Differentially expressed genes include known JAK/STAT targets *chinmo*, *Socs36E*, *dome*, and *zfh2*, which were upregulated 4.73 fold (*P* < 5.44 × 10^−23^), 1.88 fold (*P* < 4.59 × 10^−6^), 1.74 (*P* < 1.77 × 10^−6^), 1.86 fold (*P* < 2.13 × 10^−10^), respectively (Figure 2A, Table 1 and (Flaherty *et al.* 2009; Ayala-Camargo *et al.* 2013)). Other significantly upregulated genes in STAT supercompetitors were *small conductance calcium-activated potassium channel* (*SK)*, *methuselah-like 8* (*mthl8)*, long non-coding RNA *CR46123*, and the uncharacterized gene *CG30428* (Figure 2A and Table S1). We chose to impose the same cut-off and adjusted p-value in a symmetric manner to the downregulated genes (fold change ≤ 0.667 corresponding to log_2_(fold change) -0.58 and p-value < 0.05), resulting in a list of 517 downregulated genes (Figure 2A and Table S1). The most significantly downregulated gene was *pannier* (*pan*) (Figure 2A and Table S1), which we previously demonstrated was negatively regulated by JAK/STAT signaling in imaginal discs (Ekas *et al.* 2006). Importantly, neither *Myc* nor established Myc targets *nop5* and *Tif-1A* (Grewal *et al.* 2005) were upregulated in STAT supercompetitors (Table 1). However, the Myc-regulated gene *Nop60B* was upregulated 1.35 fold (*P* < 0.031) in STAT supercompetitors compared to controls (Table 1). In sum, bioinformatic analyses revealed 1004 differentially regulated genes in STAT supercompetitors compared to controls, with 487 upregulated and 517 downregulated.

**Table 1:** Expression of differentially upregulated genes in STAT supercompetitors, in Myc supercompetitors, or in both STAT and Myc supercompetitors

Gene	FC (*hop vs. gfp*)	p^adj^ value	FC (*Myc vs. gfp*)	p^adj^ value
*hop*	16.57	4.73 x 10^−180^	0.93	0.821
*Socs36E*	1.88	4.59 x 10^−6^	0.80	0.346
*chinmo*	4.74	5.44 x 10^−23^	1.48	0.0925
*dome*	1.74	1.77 x 10^−6^	0.88	0.600
*zfh2*	1.86	2.13 x 10^−10^	0.87	0.420
*Ama*	2.31	4.11 x 10^−6^	0.74	0.283
*dilp8*	3.91	8.01 x 10^−13^	1.50	0.182
*Mmp1*	1.78	0.00254	0.86	0.704
*sas*	1.59	0.00345	1.09	0.822
*edl*	2.10	0.00210	1.29	0.556
*ftz-f1*	1.82	0.00345	1.40	0.234
*ImpE1*	1.65	0.0246	0.78	0.472
*Eip75B*	1.36	0.0130	0.82	0.217
*Hr38*	2.42	2.89 x 10^−9^	1.14	0.737
*EcR*	1.46	0.0417	1.06	0.906
*Mpcp2*	1.50	0.00172	2.02	1.04 x 10^−9^
*mnd*	1.59	0.0103	1.82	0.000432
*mld*	1.62	4.10 x 10^−4^	1.52	0.00272
*Nop60B*	1.35	0.0306	1.86	1.11 x 10^−7^
*myc*	1.32	0.161	2.46	2.79 x 10^−9^
*nop5*	1.21	0.218	1.45	0.00448
*Tif-IA*	1.11	0.698	1.47	0.0210
*betaTub56D*	1.22	0.210	1.00	0.993

Legend: FC means “fold change”. p^adj^ value is the adjusted p-value.

We chose to impose the same cut-offs for the Myc supercompetitor RNA-seq, and this analysis revealed 328 differentially regulated genes, with 266 upregulated and 71 downregulated (Table S2). As expected, *Myc* was strongly upregulated, as were Myc target genes *Nop60B*, *nop5* and *Tif-1A* (Figure 2B and Table 1). Other significantly upregulated genes in Myc supercompetitors were *S-adenosylmethionine Synthetase* (*Sam-S*), *PAR-domain protein 1* (*Pdp1*), *NADH dehydrogenase (ubiquinone) 13 kD A subunit* (*ND-13A*), and *Mitochondrial phosphate carrier protein 2* (*Mpcp2*) (Figure 2B, Tables 1 and S2). The most significantly downregulated gene was *Ectoderm-expressed 3* (*Ect3*), which encodes a betagalactosidase (Figure 2B and Table S2). Neither JAK/STAT pathway components nor target genes were differentially upregulated in Myc supercompetitors (Table 1). However, the JAK/STAT target *chinmo* was increased in Myc supercompetitors with an adjusted p-value approaching significance (1.48 fold, *P* < 0.0925). Taken together, these results indicate that these transcriptome datasets accurately captures the expression profiles of JAK/STAT activation in STAT supercompetitors and of Myc mis-expression in Myc supercompetitors. There were only 41 genes differentially regulated in both types of supercompetitors, with 24 genes upregulated in both (Table S3), including *Mpcp2*, and 17 downregulated in both (Table S4).

We analyzed the differentially regulated genes in STAT supercompetitors for the presence of a Stat92E binding site in non-coding regions (see Materials and Methods). 133 significantly upregulated genes (fold change ≥ 1.5 and *P* < 0.05) in STAT supercompetitors had at least one Stat92E binding site (Table S5). These include established JAK/STAT target genes *Socs36E* with 8 binding sites and *chinmo* with 6 binding sites, as well as *Hr38* with 2 sites. *Hr38* has not been previously implicated as a possible JAK/STAT target. Of the differentially downregulated genes, 77 had at least one Stat92E binding site, including *pnr* (Table S6).

### STAT induces supercompetition by mechanisms distinct from other winners

We surveyed the differentially regulated genes for factors known to regulate winner function in various types of cell competition. Wg supercompetitors secrete Notum, a conserved secreted feedback inhibitor of Wg signaling (Vincent *et al.* 2011). However, *notum* transcripts are not significantly altered in STAT winners (Table 2). Myc supercompetitors upregulate expression of *spz*, which encodes a Toll ligand, and *Spaetzle-Processing Enzyme* (*SPE*) and *modular serine protease* (*modSP*), which encode serine proteases that cleave Spz protein into an active form (Alpar *et al.* 2018). Cleaved Spz then triggers Toll signaling in losers, which activates NFκB proteins that induce apoptosis. STAT supercompetitors did not have an increase in *spz* genes (*spz*, *spz3*, *spz4*, *spz6*), *SPE* or *modSP* (Table 2). In polarity-deficient competition, the serine protease inhibitor Serpin 5 (Spn5) is required in wild-type winners to eliminate *scrib*-deficient cells (Katsukawa *et al.* 2018). Mechanistically, Spn5 prevents the cleavage of Spz into the active form. In the absence of Spn5 secreted from wild-type winners, active Spz is produced and it triggers the growth (not death) of *scrib*-mutant cells via Toll signaling. [Note that this is the opposite result from the role of Spz-Toll in Myc supercompetition.] *Spn5* is not upregulated in STAT winners (Table 2).

**Table 2:** Genes upregulated in other winners are not differentially expressed in STAT supercompetitors

Gene	Fold Change (*hop vs. gfp*)	Adjusted p-value (*hop vs. gfp*)
*notum*	1.31	0.244
*spz*	0.79	0.504
*spz3*	1.04	0.884
*spz4*	0.90	0.829
*spz6*	1.03	0.952
*SPE*	1.01	0.987
*modSP*	1.08	0.813
*Spn5*	1.23	0.185
*Pvr*	0.96	0.855

**Figure 3:**
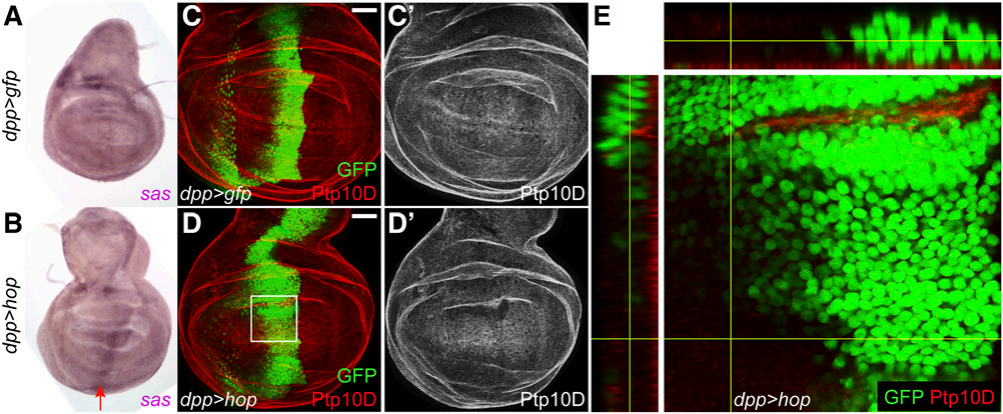
*sas* is upregulated in STAT winners but Ptp10D expression remains apical in wild-type losers. (A-B) *in situ* hybridization reveals that *sas* is expressed at moderate ubiquitous levels in a control *dpp > gfp* disc with some increased expression in the dorsal and lateral hinge in the anterior compartment (A). *sas* is upregulated along the *dpp* domain in a *dpp > hop* discs (B, arrow). At least 10 discs of each genotype were analyzed for expression pattern of the RNA probe, and the representative image of the expression pattern is shown. (C-D) Ptp10D protein (red) is expressed on the apical surface of cells in a control *dpp > gfp* (C) and a *dpp > hop* (D) disc. The *dpp* domain is marked by *UAS-gfp* (green) in C and D. (E) x-z section of the boxed region in D reveals that Ptp10D protein is not expressed at the lateral margin at the interface between STAT winners (green) and wild-type losers. Yellow lines indicate the position of x-z scan. Scale bar indicates 50 µM. Genotypes (A,C) *w/w*; *+/+*; *dpp-gal4*, *UAS-gfp/+* (B, D, E) *w/w*; *+/+*; *dpp-gal4*, *UAS-gfp/UAS-hop*.

Wild-type winners also eliminate polarity-deficient neighbors by the Sas-Ptp10D system (Yamamoto *et al.* 2017) and Pvr-dependent engulfment (Ohsawa *et al.* 2011). Stranded at second (Sas) is a transmembrane protein that acts as a ligand for the transmembrane phosphatase Ptp10D (Schonbaum *et al.* 1992; Lee *et al.* 2013). Wild-type winners require *sas* to eliminate polarity-deficient losers (Yamamoto *et al.* 2017). At the interface between wild-type winners and *scrib*-mutant cells, both Sas and Ptp10D relocalize from the apical domain to the lateral domain (Yamamoto *et al.* 2017). *sas* transcripts are significantly upregulated in STAT supercompetitors (1.59 fold, *P* < 0.00345) but not in Myc supercompetitors (Table 1), and the *sas* gene has one Stat92E binding site (Table S5). We used *in situ* hybridization to monitor *sas* transcripts in *dpp > hop* discs compared to *dpp > gfp* controls. *sas* mRNA is expressed at low levels in control wing discs, with the exception of a couple of patches at the notum-hinge interface (Figure 3A). *sas* mRNA is upregulated in STAT supercompetitors residing in the hinge and pouch (Figure 3B, arrow). In both control and *dpp > hop* discs, Ptp10D protein is localized to the apical domain as expected (Figure 3C,D). Importantly, in *dpp > hop* discs Ptp10D is **not** localized to the lateral interface between STAT winners and wild-type losers (Figure 3E), suggesting that the Sas-Ptp10D system does not function in JAK/STAT-dependent cell competition. In polarity-deficient competition, wild-type winners also upregulate Pvr, the *Drosophila* PDGF/VEGF receptor. However, Pvr is not changed in STAT winners compared to controls (Table 2). Taken together, these observations suggest that STAT supercompetitors are distinct from other kinds of winners.

### STAT winners upregulate Duox and ROS

**Figure 4:**
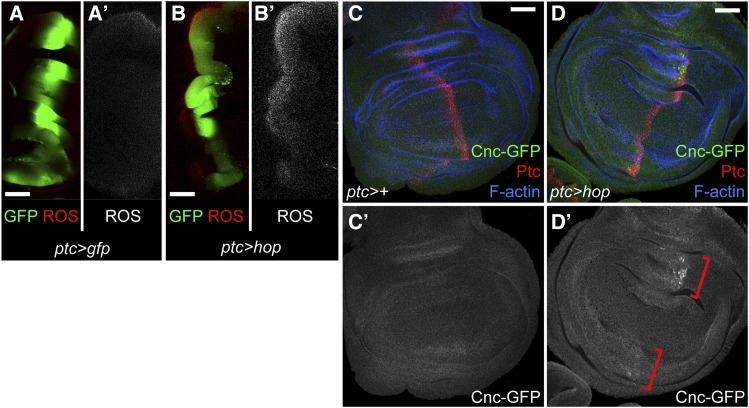
ROS and Nrf2 are upregulated in STAT winners. (A-B) In a control *ptc > gfp* disc (A), cells in the *ptc* domain (labeled by GFP, green) have low levels of ROS as assessed by CellROX Deep Red Reagent (red) (A). In a *ptc > hop* disc (B), STAT winners (labeled by GFP, green) generated in the *ptc* domain have elevated levels of ROS (red) along the entire *ptc* domain (B). (C-D) Cnc-GFP, encoded by a bacterial artificial chromosome under endogenous regulatory sequences, is expressed at low levels in a control *ptc-gal4* disc (C). Cnc-GFP is upregulated in STAT winners, particularly in the dorsal and ventral hinge (D, brackets). Ptc is red. Phalloidin, which marks F-actin, is in blue. Scale bar indicates 50 µM. Genotypes (A) w/w; *ptc-gal4*, *UAS-gfp/+*; *+/+* (B) w/w; *ptc-gal4*, *UAS-gfp/+*; *UAS-hop/+* (C) w/w; *ptc-gal4/ PBac(cnc-EGFP.S)VK00037: +/+* (D) w/w; *ptc-gal4/ PBac(cnc-EGFP.S)VK00037*; *UAS-hop/+*.

Dual oxidase (Duox) is an enzyme that produces extracellular reactive oxygen species (ROS) by catalyzing the transmembrane electron transfer from the intracellular NADPH-FAD electron donors to the extracellular space, reducing oxygen to superoxide or hydrogen peroxide (De Deken *et al.* 2014). In *Drosophila*, the sole *Duox* gene plays a central role in gut immunity, where its upregulation at the gene and protein level is required for innate immune response that eliminate infectious bacteria (Ha *et al.* 2009a; Ha *et al.* 2009b). *Duox* is significantly increased in STAT supercompetitors (1.77 fold, *P* < 0.00238), while genes encoding other ROS-producing enzymes like *NADPH oxidase* (*Nox*) are not (Table 3). If Duox expression is increased in STAT supercompetitors, ROS should be increased in STAT winners. To test this, we generated STAT winners in the anterior domain of the wing disc by expressing *UAS-hop* with *ptc-gal4*, a driver expressed in anterior cells located closest to the anterior-posterior boundary. We monitored ROS using a protocol established by the Serras lab (Santabárbara-Ruiz *et al.* 2015). Indeed, we find that ROS are specifically increased in the *ptc* domain of *ptc > hop* wing discs (Figure 4B). By contrast, ROS are not observed in control *ptc > gfp* discs (Figure 4A).

**Table 3:** Expression of genes encoding ROS-generating or anti-oxidant factors in STAT supercompetitors

Gene	Fold Change (*hop vs. gfp*)	Adjusted p-value (*hop vs.* gfp)
*cnc*	1.30	0.0618
*Nox*	0.684	0.0957
*Keap1*	0.68	0.00726
*GstD5*	2.45	0.000933
*GstD6*	2.07	0.0186
*GstD3*	2.05	0.000392
*GstD4*	2.04	0.0167
*GstD10*	1.70	0.0138
*GstD1*	1.56	0.000658
*Ugt86Di*	2.31	1.23 x 10^−6^
*Ugt86Da*	1.71	0.00602
*Cyp18a1*	3.21	4.26 x 10^−5^
*Cyp4aa1*	1.98	0.0222
*Cyp9h1*	1.88	0.0669

### STAT supercompetitors upregulate Nrf2

Our results indicate that STAT winners reside in an oxidizing environment caused by ROS production. We hypothesize that to protect themselves from this environment, STAT winners must upregulate a mild anti-oxidant response. Nrf2 (called Cap-n-collar (Cnc) in *Drosophila*) is a transcription factor that regulates numerous genes controlling oxidant homeostasis (Ma 2013). Under basal conditions, Nrf2 is sequestered in the cytoplasm through physical interactions with Keap1, which promotes Nrf2’s proteasomal degradation. Oxidants activate Nrf2 by modifying critical cysteine thiols on Keap1. This liberates Nrf2 to translocate to the nucleus, bind to anti-oxidant response elements and induce target gene expression, including Glutathione S-transferases (GSTs), the main cytosolic reducing agents (Taguchi *et al.* 2011; Sies *et al.* 2017). STAT winners have a transcriptional signature of an anti-oxidant response with a moderate but significant increase in *cnc* (1.30 fold, *P* < 0.0618) and a moderate but significant decrease in *Keap1* (0.678 fold and *P* < 0.00726) (Table 3). These transcriptional changes should increase Nrf2 protein and decrease its inhibitor, resulting in a protective anti-oxidant response. Additionally, numerous Nrf2 target genes are significantly increased in STAT winners compared to controls (Table 3), include those encoding six cytosolic GSTs, two UDP-glucosyltransferases (Ugt), which reduce hydrophobic molecules, and three cytochrome P450s (Cyp), which reduce a large variety of substrates (Bock 2003; Coon 2005). To validate the increased *cnc* expression in STAT winners, we mis-expressed *UAS-hop* in the *ptc* domain in a genetic background that carried a bacterial artificial chromosome containing *cnc* under the control of endogenous regulatory elements C-terminally tagged with *gfp*. We find that Cnc-GFP is upregulated in STAT winners (Figure 4D, brackets), most strongly in the dorsal and ventral hinge, which are the sites of highest endogenous JAK/STAT signaling in third instar wing discs (Bach *et al.* 2007; Ayala-Camargo *et al.* 2013). By contrast, Cnc-GFP is not observed in control *ptc>+* discs (Figure 4C).

### Ecdysone signaling is upregulated in STAT supercompetitiors

Of the genes in Flybase that have been reported to part of the ecdysone pathway (Ihry and Bashirullah 2014; Jiang *et al.* 2018), 14 are differentially regulated in STAT supercompetitors. This group includes these upregulated genes, *Eip75B*, *ftz-f1*, *EcR*, *ImpE1*, *Cyp18a1*, *Ecdysone Importer* (*EcI*) (Flybase: *Oatp74D*), *Iswi*, *swi2*, *E(bx)*, *hid* and *rpr*, and these downregulated genes, *Blimp-1*, *let-7-C* and *DopEcR* (Tables 4 and S1). Interestingly, several of these genes have STAT binding sites including *ImpE1*, *ftz-f1* and *Blimp-1* with 1 site each (Tables S5 and S6). Additionally, *Eip75B* has 4 Stat92E binding sites and *EcR* has 1, but neither was included in Table S5 because the fold change was below the 1.5 fold cut-off for upregulated genes. STAT winners could have increased ecdysone signaling compared to control *dpp > gfp* cells because the gene encoding the transporter required for ecdysone uptake *EcI* is differentially upregulated 1.68 fold (*P* < 7.1 × 10^−5^) in STAT supercompetitors (Tables 4 and S1 and (Okamoto *et al.* 2018)). Since the *EcI* locus does not contain STAT binding sites, it remains unclear how *EcI* is upregulated in STAT winners, but it may be an indirect target.

**Table 4:** Expression of genes in the ecdysone pathway or ecdysone responsive genes in STAT supercompetitors

Gene	Fold Change (*hop vs. gfp*)	Adjusted p-value (*hop vs. gfp*)
*Eip75B*	1.36	0.0131
*ftz-f1*	1.82	0.00345
*EcR*	1.45	0.0417
*ImpE1*	1.66	0.0246
*Cyp18a1*	3.21	4.262 x 10^−5^
*Oatp74D*	1.69	7.191 x 10^−5^
*Iswi*	1.34	0.0106
*swi2*	1.73	0.0478
*E(bx)*	1.33	0.0180
*hid*	1.50	0.0164
*rpr*	2.05	0.00657
*Blimp-1*	0.53	0.00532
*let-7-C*	0.50	0.0249
*DopEcR*	0.61	0.0722

**Figure 5:**
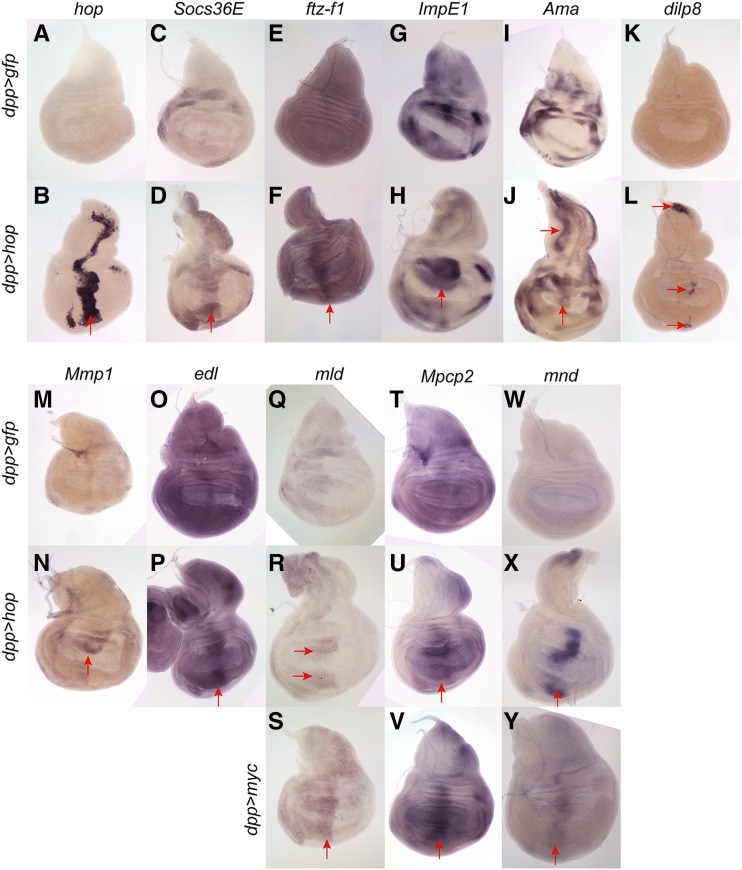
Validation of genes upregulated in STAT supercompetitors. *in situ* hybridization for genes upregulated in STAT supercompetitors (A-P) and genes upregulated in both STAT and Myc supercompetitors (Q-Y). At least 10 discs of each genotype were analyzed for expression pattern of each RNA probe, and the representative image of the expression pattern is shown. (A-B) *hop* is expressed at low levels in control (*dpp > gfp*) discs (A) but is upregulated along the *dpp* domain in *dpp > hop* discs (B, arrow). (C-D) *Socs36E* is expressed in several distinct patches in the dorsal, lateral and ventral hinge in a control disc (C) but is increased along the *dpp* domain in a *dpp > hop* disc (D, arrow). (E-F) *ftz-f1* is expressed at low levels in a control disc (E) but is induced in STAT supercompetitors located in the *dpp* domain (F, arrow). (G-H) *ImpE1* is expressed in numerous discrete patches in a control (*dpp > gfp*) disc (G) and is upregulated in the dorsal hinge in a *dpp > hop* disc (H, arrow). (I-J) *Ama* is expressed in many patches of cells in the hinge and notum in a control disc (I) but is upregulated along the *dpp* domain in a *dpp > hop* disc (J, arrows). (K-L) *dilp8* is expressed at low levels in a control (*dpp > gfp*) disc (K) but is induced in several distinct regions in the notum, hinge and pouch in a *dpp > hop* disc (L, arrows). (M-N) *Mmp1* is expressed at low levels in a control (*dpp > gfp*) disc (M) but is upregulated within the dorsal hinge in a *dpp > hop* disc (N, arrow). (O-P) *edl* is expressed robustly and ubiquitously in a *dpp > gfp* disc (K) but is upregulated in along the *dpp* domain in a *dpp > hop* disc (L, arrow). (Q-S) *mld* is expressed at moderate levels in anterior cells in a control wing disc (Q). *mld* is upregulated in both the dorsal and ventral hinge in a *dpp > hop* disc (R, arrows) and along the entire *dpp* domain in a *dpp > Myc* disc (S, arrow). (T-V) *Mpcp2* is expressed robustly in a control wing disc (T). *Mpcp2* is upregulated in the *dpp* domain in a *dpp > hop* (U, arrow) and a *dpp > Myc* disc (V, arrow). (W-Y) *mnd* is expressed at low levels in a control wing disc (W). *mnd* is upregulated in the *dpp* domain in a *dpp > hop* (X, arrow) and a *dpp > Myc* disc (Y, arrow). Genotypes (A, C, E, G, I, K, M, O, Q, T, W) *w/w*; *+/+*; *dpp-gal4*, *UAS-gfp/+* (B, D, F, H, J, L, N, P, R, U, X) *w/w*; *+/+*; *dpp-gal4*, *UAS-gfp/UAS-hop* (S, V, Y) *w/w*; *+/+*; *dpp-gal4*, *UAS-gfp/UAS-Myc*.

We used *in situ* hybridization to validate some ecdysone pathway genes upregulated in STAT supercompetitors. As a proof of principle, we first assessed the expression pattern of *hop* (the mis-expressed gene in the STAT RNA-seq) and *Socs36E*, the best characterized JAK/STAT target gene (Bach *et al.* 2007). *hop* mRNA was expressed at low levels in control wing discs (Figure 5A) and, as predicted, was upregulated along the entire *dpp* domain in *dpp > hop* discs (Figure 5B, arrow). *Socs36E* mRNA is restricted to the presumptive hinge domain in control discs (Figure 5C). In *dpp > hop* discs, *Socs36E* mRNA is ectopically induced along the *dpp* stripe (Figure 5D, arrow).

Having proven the efficacy of *in situ* for validating differentially expressed genes in the RNA-seq, we next turned our attention to some ecdysone pathway genes. Ftz-f1 is a nuclear hormone receptor expressed normally at high levels in mid-prepupal stages, when it acts as a critical competence factor for the response to the late pupal ecdysone pulse (Woodard *et al.* 1994; Broadus *et al.* 1999). *ftz-f1* is significantly upregulated in STAT winners (1.83 fold, *P* < 0.00345) but not in Myc winners (Table 1), and, as noted above, *ftz-f1* has 1 Stat92E binding site (Table S5). Consistent with its induction in mid-pupariation, *ftz-f1* mRNA is expressed at low levels in control third instar wing discs (Figure 5E). It is upregulated in STAT supercompetitors along most of the *dpp* stripe (Figure 5F, arrow). An early ecdysone response gene *ImpE1* encodes a protein similar to a low-density lipoprotein receptor (Natzle *et al.* 1988; Natzle 1993). *ImpE1* is upregulated 1.6 fold (*P* < 0.0246) in STAT supercompetitors but not in Myc supercompetitors (Table 1), and the gene has 1 Stat92E binding site (Table S5). *ImpE1* mRNA is expressed in several distinct patches in a control third instar wing disc (Figure 5G), and it is upregulated in STAT supercompetitors located in the dorsal hinge in *dpp > hop* discs (Figure 5H, arrow).

### Validation of other genes differentially regulated in STAT supercompetitors

Because STAT supercompetitors non-autonomously cause the death of wild-type neighboring cells, we next examined differentially expressed genes that encode secreted or transmembrane proteins. Amalgam (Ama) is a secreted Ig-domain containing protein that mediates cell-cell-adhesion (Seeger *et al.* 1988; Fremion *et al.* 2000; Zeev-Ben-Mordehai *et al.* 2009; Özkan *et al.* 2013). It is significantly upregulated (2.32 fold, 4.11 × 10^−6^) in STAT supercompetitors but not in Myc supercompetitors (Table 1). *Ama* mRNA is observed in numerous discrete domains in the presumptive hinge and notum in control discs (Figure 5I). It is induced in the *dpp* domain in *dpp > hop* discs (Figure 5J, arrows). *Drosophila* insulin-like peptide 8 (Dilp8, Flybase Ilp8) is a relaxin-like protein that controls developmental timing by regulating the release of ecdysone by neuroendocrine cells in the brain (Colombani *et al.* 2012; Garelli *et al.* 2012; Colombani *et al.* 2015; Garelli *et al.* 2015; Vallejo *et al.* 2015). *dilp8* transcripts are significantly upregulated (3.91 fold, *P* < 8.01 × 10^−13^) in STAT supercompetitors but not in Myc supercompetitors (Table 1). However, despite the upregulation of *dilp8* transcripts in STAT winners, *dpp > hop* animals are not developmentally delayed (see above), possibly because the amount of ectopic *dilp8* in *dpp > hop* discs is insufficient to activate Lgr3-expressing neurons in the brain (Colombani *et al.* 2015; Garelli *et al.* 2015; Vallejo *et al.* 2015). *dilp8* is present at low levels in control wing discs (Figure 5K) and is upregulated in several discrete areas along the *dpp* stripe in *dpp > hop* discs (Figure 5L, arrows). Matrix metalloproteinase 1 (Mmp1) is a secreted protease that cleaves substrates in the extracellular matrix and regulates tissue remodeling and wound healing (Page-McCaw *et al.* 2003). *Mmp1* transcripts are augmented 1.78 fold (*P* < 0.00254) in STAT winners but not in Myc winners (Table 1). *Mmp1* mRNA is expressed at low levels in a control third instar wing disc (Figure 5M), consistent with a prior report (Page-McCaw *et al.* 2003), and it is increased in STAT supercompetitors located in the dorsal hinge (Figure 5N, arrow), the hinge being the site of highest endogenous activity of the JAK/STAT pathway.

We also validated upregulated genes in STAT supercompetitors that encode in intracellular proteins. ETS-domain lacking (Edl) acts downstream of MAPK to promote Epidermal growth factor receptor signaling (Baker *et al.* 2001; Tootle *et al.* 2003). *edl* transcripts are significantly upregulated in STAT supercompetitors (2.10 fold, *P* < 0.00209) but not in Myc supercompetitors (Table 1). *edl* mRNA is observed at moderate levels throughout the wing (Figure 5O) and is upregulated in STAT supercompetitors along the *dpp* domain (Figure 5P, arrow).

Finally, we validated genes differentially upregulated in both STAT and Myc supercompetitors. *molting defective* (*mld*) encodes a nuclear, zinc-finger domain protein required for ecdysone biosynthesis (Neubueser *et al.* 2005). *mld* is significantly increased in both STAT and Myc supercompetitors compared to controls (1.61 fold, *P* < 0.0004 for STAT and 1.61 fold, *P* < 0.0004 for Myc, Table 1). *mld* mRNA is expressed at low levels in a control third instar wing disc, higher in the anterior domain than the posterior (Figure 5Q). *mld* is increased in STAT supercompetitors located in the dorsal and ventral hinge (Figure 5R, arrows) and in Myc supercompetitors located in the *dpp* domain in the pouch (Figure 5S, arrow). As noted above, *Mpcp2* is significantly upregulated in both STAT and Myc supercompetitors (1.50 fold, *P* < 0.00172 for Hop; 2.02 fold, *P* < 1.036 × 10^−10^ for Myc, and Table 1). Mpcp2 is a nuclear-encoded, mitochondrial inner membrane transporter that facilitates the movement of metabolites, nucleotides and cofactors across this mitochondrial membrane (Palmieri 2013). *Mpcp2* is expressed at moderate and fairly uniform levels in a control third instar wing disc (Figure 5T) and is upregulated in *dpp > hop* and *dpp > Myc* discs in the *dpp* domain of the pouch and hinge (Figure 5U and V, arrows). *minidiscs* (*mnd*) encodes a leucine amino acid transporter (Martin *et al.* 2000; Reynolds *et al.* 2009). *mnd* is significantly upregulated in both STAT and Myc supercompetitors (1.59 fold, *P* < 0.0103 for Hop; 1.82 fold, *P* < 0.000432 for Myc and Table 1). *mnd* is expressed a low level in a control disc (Figure 5W) but is upregulated along the *dpp* stripe in *dpp > hop* and *dpp > Myc* discs (Figure 5X and 5Y, arrows).

### Establishing a quantitative assay for supercompetition

**Figure 6:**
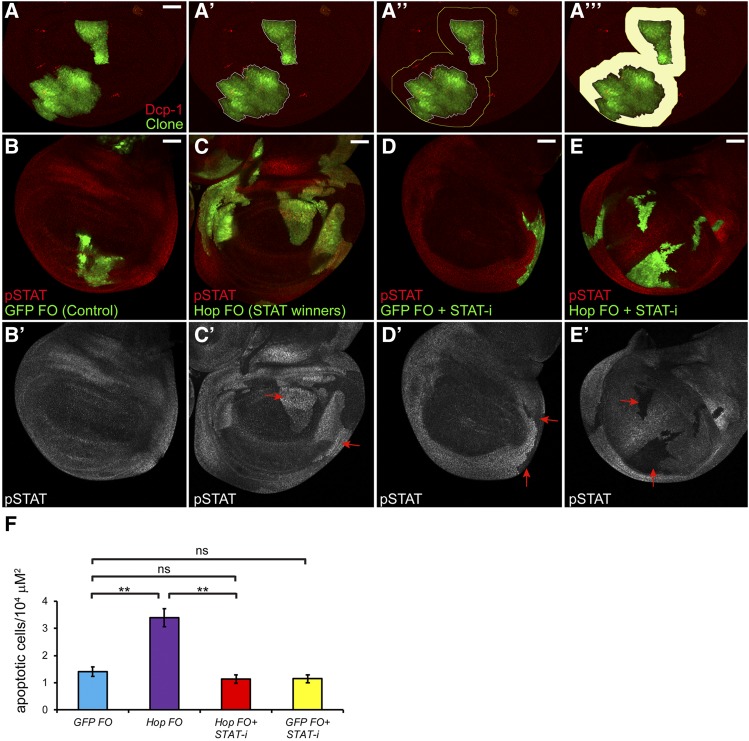
Quantitative assay for supercompetition. (A) A representative wing disc with Hop flip-out clones (green) labeled with Dcp-1 (red) to mark apoptotic cells. We used Image J to outline the Hop flip-out clones (A’) and then drew a second line 10-cell diameters away from the clone boundary (A”). We counted the number of dying cells within the shaded area (A’”). (B) Control GFP flip-out (*GFP FO*) clones (green) do not have activated STAT (red, labeled pSTAT). (C) Hop flip-out (*Hop FO*) clones (green) ectopically activate STAT (C’, arrows). (D) Control GFP flip-out clones expressing a *UAS-Stat92E* RNAi construct (green, labeled *GFP FO + STAT-i*) display autonomous loss of STAT (D’, arrows). (E) Hop flip-out clones expressing a *UAS-Stat92E* RNAi construct (green, labeled *Hop FO + STAT-i*) display autonomous loss of STAT (E’, arrows). Note: in B-E, the clones were not induced in timed embryo collections and hence they are of varying sizes. Scale bar indicates 50 µM. (F) Graph displays the number of apoptotic (Dcp-1-positive) wild-type neighbors per unit area (10^4^ µM^2^). There are significantly more apoptotic neighbors of STAT supercompetitors (*Hop FO*, purple bar) than control clones (*GFP FO*, blue bar). When *Stat92E* is depleted from STAT supercompetitors (*Hop FO + STAT-i*, red bar), there is a significant reduction in the number of apoptotic wild-type neighbors. By contrast, depletion of *Stat92E* from control clones (*GFP FO + STAT-i*, yellow bar) does not alter the viability of wild-type neighbors. ** *P* < 0.001; “ns” is not significant. Genotypes (A, C) *y*, *w*, *hs-flp^122^/+*; *act > y+>gal4*, *UAS-gfp/UAS-Dcr-2*; *UAS-hop/+* (B) *y*, *w*, *hs-flp^122^/+*; *act > y+>gal4*, *UAS-gfp/UAS-Dcr-2*; *+/+* (D) *y*, *w*, *hs-flp^122^/+*; *act > y+>gal4*, *UAS-gfp/UAS-Dcr-2*; *UAS-Stat92E^HMS00035^/+* (E) *y*, *w*, *hs-flp^122^/+*; *act > y+>gal4*, *UAS-gfp/UAS-Dcr-2*; *UAS-hop/UAS-Stat92E^HMS00035^* (F) *y*, *w*, *hs-flp^122^/+*; *act > y+>gal4*, *UAS-gfp/UAS-Dcr-2*; *+/+ y*, *w*, *hs-flp^122^/+*; *act > y+>gal4*, *UAS-gfp/UAS-Dcr-2*; *UAS-hop/+ y*, *w*, *hs-flp^122^/+*; *act > y+>gal4*, *UAS-gfp/UAS-Dcr-2*; *UAS-Stat92E^HMS00035^/+ y*, *w*, *hs-flp^122^/+*; *act > y+>gal4*, *UAS-gfp/UAS-Dcr-2*; *UAS-hop/UAS-Stat92E^HMS00035^*.

We developed an assay to quantify supercompetitor-induced apoptosis of wild-type neighbors (see Materials and Methods). We generated random clones mis-expressing GFP alone (*i.e.*, control clones) or GFP plus Hop (*i.e.*, STAT supercompetitor clones) precisely at 48 hr AED. We dissected wing discs 72 hr later (at 120 hr AED). After scanning the samples on a confocal microscope, we used Image J to outline the clone (Figure 6A-A’) and then to draw another line representing 10 cell-diameters from the clone boundary (Figure 6A”-A”’). We then counted the number of apoptotic wild-type cells within the area between the two lines. There were significantly more dead wild-type cells neighboring STAT supercompetitors than those neighboring control clones (Figure 6F, compare purple to blue bar, *P* < 0.001). Clonal mis-expression of Hop induces STAT activation in a cell-autonomous manner (Figure 6C), whereas clonal mis-expression of GFP does not (Figure 6B). To prove that the competitive stress inflicted by supercompetitors was due to increased JAK/STAT pathway activity, we depleted *Stat92E* from both types of clones. This resulted in a robust autonomous decrease in STAT antibody reactivity in both types of clones (Figure 6D,E). Depletion of *Stat92E* from STAT supercompetitors substantially reduced their competitive properties, as assessed by significantly fewer apoptotic wild-type neighbors (Figure 6F, compare purple bar to red bar, *P* < 0.001). In fact, Hop clones depleted for *Stat92E* were now indistinguishable from control clones with respect to neighbor death (Figure 6F, no significant difference between the red and blue bars). As expected, depletion of *Stat92E* from control clones did not affect their wild-type neighbors (Figure 6F, no significant difference between yellow and blue bars).

## Discussion

Here we report the transcriptional profiling of highly purified STAT or Myc supercompetitors from wing imaginal discs. We demonstrate that the transcriptional profiles of these two type of competitors are largely distinct, with only 41 genes that are differentially regulated in both data sets. Our interest lies in identifying JAK/STAT target genes that regulate the competitive abilities of STAT supercompetitors. Using a combination of protein traps, immunofluorescence and *in situ* hybridization, we validated numerous upregulated genes in STAT supercompetitors, including several genes encoding secreted or transmembrane proteins. Our characterization of differentially regulated genes in STAT winners demonstrates that they have increased ROS generation, and, presumably as a result of this, an anti-oxidant response. Recent work has proposed that an anti-oxidant response is a hallmark of cells heterozygous for a ribosomal gene that can be outcompeted when confronted by wild-type cells (Kucinski *et al.* 2017). Our work demonstrates that STAT supercompetitors have a similar signature, suggesting that the anti-oxidant response is not a universal marker of less fit cells. In the future, we will need to determine whether *Duox* upregulation causes the anti-oxidant response and whether *Duox* and *cnc* are required for the competitive properties of STAT winners.

We report here that the established Jun N-terminal kinase (JNK) target *Mmp1* is significantly upregulated in STAT winners, and this result suggests that JNK signaling in increased in STAT winners. Prior work has shown that *ftz-f1* can be upregulated by JNK in imaginal discs (Külshammer *et al.* 2015). Consistent with increased JNK signaling in STAT winners, *ftz-f1* is increased in STAT winners compared to control cells. JNK is required in wild-type winners to eliminate polarity-deficient losers (Ohsawa *et al.* 2011), and in the future, we will need to determine if JNK signaling facilitates the competitive properties of STAT winners. We will also need to address how JNK signaling is activated in STAT winners, particularly whether JNK is activated downstream of Duox or ROS generation in these cells.

STAT supercompetitors have increased ecdysone signaling and this is not shared with Myc supercompetitors. We suggest that the significantly increased expression of *EcI*, the transporter required for ecdysone movement into cells, may underlie the heightened ecdysone responses in STAT supercompetitors, but future work will be needed to test this model. Recent work has shown that ecdysone signaling promotes growth of imaginal discs (Herboso *et al.* 2015; Neto *et al.* 2017). Work from the Casares and Aerts labs has shown that mis-expressing transcription factors Homothorax (Hth) and Teashirt (Tsh) in the early eye disc causes a significant increase in *ftz-f1* and a significant decrease in *Hormone receptor 3* (*Hr3*, also called *Dhr3* or *Hr46*) and in *Blimp-1*. They reported that changes in these genes promote proliferation of undifferentiated eye disc progenitors (Neto *et al.* 2017). STAT winners share some elements of this Hth+Tsh profile, as they significantly upregulate *ftz-f1* and significantly downregulate *Blimp-1* (Table 4). However, *Hr3* is not differentially expressed in these cells. Future work will be needed to address if *ftz-f1* and *Blimp-1* are required for the proliferation and/or growth of STAT winners.

Eip75B is a heme-binding nuclear receptor that acts as a transcriptional repressor by inhibiting Hr3 (Reinking *et al.* 2005). When nitric oxide, the product of the sole *Drosophila* nitric oxide synthase (Nos), binds to the heme center, the interaction between Eip75B and Hr3 is curtailed. This liberates Hr3 to function as a transcriptional co-activator and induce expression of target genes, particularly *ftz-f1* (Caceres *et al.* 2011). The fact that *Eip75B* and *ftz-f1* transcripts are both significantly upregulated in STAT supercompetitors suggests that nitric oxide levels are low in these cells. Although confirmation of this awaits the results of future work, it is intriguing to note that *Nos* is significantly downregulated (0.501 fold change, *P* < 0.000954) in STAT winners (Table S1). It is also interesting to note that Eip75B is proposed to function as a redox sensor because the oxidation state of the heme center dictates whether it can interact with its heterodimeric partner Hr3. It will be important to determine whether the anti-oxidant response in STAT winners impacts the Eip75B heme center.

Taken together, our transcriptomic data indicate that STAT winners are distinct from other kinds of winners. This in turn supports the concept that there are multiple types of cell competition, as opposed to a universal one, with different triggers and effectors. These transcriptomes should be valuable resources for others in the field of cell competition.
